# Vitamin D and biomarkers of inflammation and oxidative stress among pregnant women: a systematic review of observational studies

**DOI:** 10.1186/s12865-023-00577-w

**Published:** 2023-10-27

**Authors:** Soudabe Motamed, Razieh Anari, Somayeh Motamed, Reza Amani

**Affiliations:** 1 Asadabad School of Medical Sciences, Asadabad, Iran; 2 National Nutrition and Food Technology Research Institute, Faculty of Nutrition Sciences and Food Technology, Shahid Beheshti University of Medical Sciences, Tehran, Iran; 3 School of Medicine, Tehran University of Medical Sciences, Tehran, Iran; 4 Department of Clinical Nutrition, School of Nutrition and Food Sciences, Isfahan University of Medical Sciences, Isfahan, Iran

**Keywords:** 25-hydroxyvitamin D, Vitamin D, Inflammation, Oxidative stress, Pregnancy, Systematic review

## Abstract

**Objective:**

This systematic review aimed to map the evidence evaluated the relationship between vitamin D and redox and inflammatory status during gestation.

**Methods:**

Three databases (PubMed/MEDLINE, Scopus, and Web of Science (WoS)) and reference list of included documents were searched for related observational studies published until 2nd October 2023. To determine the quality of the selected observational studies, the Newcastle-Ottawa Scale (NOS) was used.

**Results:**

After a primary search of three databases, 19492records were appeared. When duplicates and irrelevant documents were removed, 14 articles were found to have eligible criteria. The design of the identified studies was cross-sectional, case-control and cohort. Evidence showed an adverse association between 25(OH)D and the biomarkers of inflammation, such as high-sensitivity C-reactive protein (hs-CRP), Interleukin-1beta (IL-1β), Interleukin-6 (IL-6), and tumor necrosis factor- alfa (TNF-α) during pregnancy. On the contrary, some studies represented that 25(OH)D positively correlated with hs-CRP in the cord blood. One study suggested a direct association between serum concentrations of 25(OH)D and Interleukin-8 (IL-8), macrophage inflammatory protein (MIP), and TNF-α levels in mothers with gestational diabetes mellitus (GDM). A case-control study showed that lower serum concentration of 25(OH)D positively correlated with total antioxidant capacity (TAC) levels in participants.

**Conclusions:**

Evidence confirmed the supposition of the direct relationship between vitamin D levels and biomarkers with anti-inflammatory and anti-oxidative properties. However, the Existence of inconsistent evidence confirms the need for further studies in mothers with GDM and hypertensive disorders.

**PROSPERO registration code:**

CRD42020202600.

**Supplementary Information:**

The online version contains supplementary material available at 10.1186/s12865-023-00577-w.

## Introduction

Vitamin D as a fat-soluble vitamin has many substantial functions in the body, such as the cells proliferation and differentiation, immunity regulation, and inflammatory and oxidative stress modulation in addition to its classical role regarding bone and dental health [1–4]. Vitamin D can also transfer through placenta and affect pregnancy outcomes via implementing changes in inflammatory and redox status [5–8].

Balanced levels of cytokines regulate pregnancy and parturition, while excessive amounts of cytokines with pro-inflammatory properties (e.g., IL-1β, IL-6, and TNF-α), were associated with GDM, hypertensive disorders of pregnancy (HDP), preterm birth, and fetal loss [9, 10]. Therefore, dysregulation of the cytokines can adversely influence pregnancy and increase pregnancy complications [11].

Similarly, the generation of reactive oxygen species (ROS) during pregnancy contributes to some physiological processes, such as embryo implantation. However, excessive ROS production and oxidative stress can lead to the disruption of developmental processes and impaired function of the placenta and consequently, the occurrence of pregnancy complications, like GDM, intra uterine growth restriction (IUGR), and pregnancy loss [12, 13]. Therefore, providing a balanced production of oxidants and antioxidants (redox status) throughout pregnancy is critical [13]. Documents suggested that sufficient levels of vitamin D is related to minimal oxidative stress and proper function of mitochondria and the endocrine system, that result in lower risk of adverse pregnancy outcomes [14]. Pregnant women have a high possibility for vitamin D deficiency due to the enhanced physiological requirements [5]. A few observational studies have been conducted to reveal the relationship between the levels of vitamin D and biomarkers of inflammation and oxidative stress during gestation. However, the mentioned relationship has not been assessed through systematic reviews during pregnancy. Therefore, the present systematic review aimed to collect and summarize the evidence that assessed the association between the status of vitamin D and inflammation and oxidative stress in pregnant women.

## Materials & methods

The Preferred Reporting Items for Systematic Reviews and Meta-analyses (PRISMA) standard was applied for writing this systematic review. The study protocol registration code is CRD42020202600 (available at: https://www.crd.york.ac.uk/prospero/display_record.php?ID=CRD42020202600).

### Search strategy

Three databases, including PubMed/MEDLINE, Scopus, and WoS, and reference list of included documents were used for a comprehensive search of literatures published from onset to 2nd July October 2023with no language restriction.

A combination of the following terms was searched: pregnancy, gestation, child bearing, gravidity, intrauterine pregnancy, labor presentation, pregnancy maintenance, pregnancy trimesters, and vitamin D. The supplemental information contains the details of the search strategy.

### Eligibility criteria

**Inclusion criteria***Population*: Pregnant women and their infant cord blood; *Exposure*: Blood levels of 25(OH)D; *Outcomes*: Biomarkers of inflammation, such as high-sensitivity C-reactive protein (hs-CRP), TNF-α, transforming growth factor-beta (TGF-β), interferon-gama (IFN-γ ), IL-1β, IL-4, IL-6, IL-7, IL-8, IL-10, IL-13, and/or oxidative stress markers, such as malondialdehyde (MDA), TAC, glutathione (GSH), and superoxide dismutase (SOD); *study design*: observational studies (cross-sectional, case-control and cohort studies), and baseline data of randomized control trials (RCTs).

**Exclusion criteria**

1. Clinical trials, animal studies, and in vitro studies.
2. Studies without reports on the levels of 25(OH)D and at least one of the biomarkers of inflammation or oxidative stress.
3. Studies with determination of the interested outcomes just in infants and not in their mothers.

### Study selection

Titles and abstracts of the records that retrieved were read by a reviewer (SM1) in order to find relevant documents. Then, two reviewers checked the full text of the included articles for their eligibility (SM1 & RA). When two reviewers came to disagreement, they resolved it by discussion and thereafter, by counselling a third expert reviewer (ReA).

### Data extraction

Two independent reviewers (SM1 & SM2) extracted the required information, such as first author, journal, date of publication, chronological and gestational age of mothers, anthropometric indices (BMI, etc.), study design, sample size, and desired outcomes (concentrations of 25(OH)D and biomarkers inflammation and oxidative stress), and reported effect sizes with their intervals.

### Risk of bias assessment

The selected articles were reviewed by two independent investigators (SM1 and RA) for risk of bias or quality assessment by using the Newcastle-Ottawa Scale (NOS) that judges the quality through three main domains including selection, comparability, and exposure/outcome, and uses “star system” for scoring. The number of stars in each domain determines the final quality. The final quality ranked “Very Good” (≥ 5 points in cross-sectional studies or 7–8 points in cohort and cross-sectional studies), “Good” (4 in Cross-sectional or 5–6 in cohort and cross-sectional studies), “Satisfactory” (3 in cross-sectional studies or 4 in cohort and cross-sectional studies), or “Unsatisfactory”(0–2 points in cross-sectional studies or 0–3 points in cohort/cross-sectional studies) [15]. A third expert reviewer (ReA) was called for final decision in case of any disagreement between the reviewers.

## Results

**Fig. 1:**
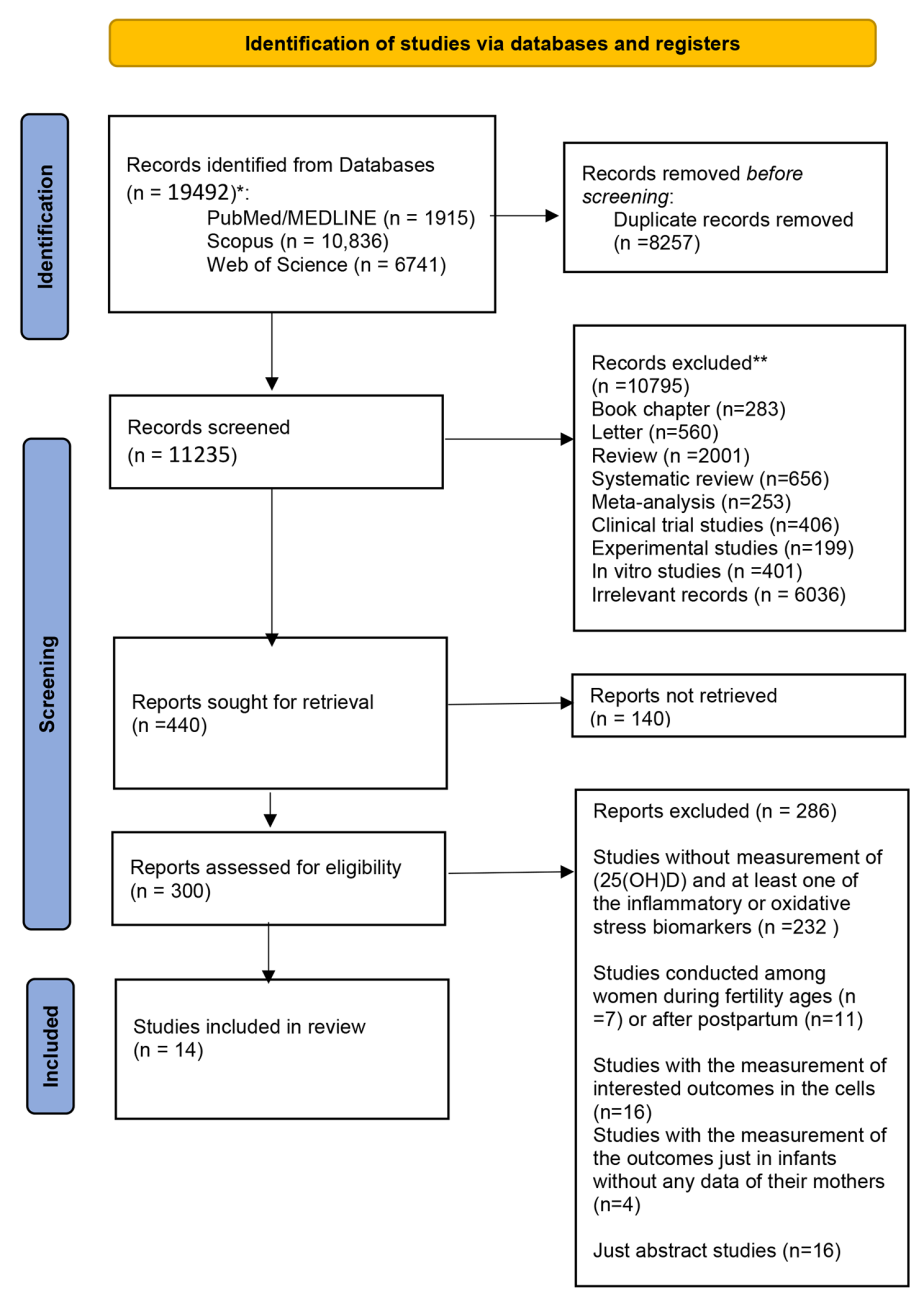
Vitamin D and biomarkers of inflammation and oxidative stress among pregnant women: a systematic review of observational studies

Figure 1 indicates that 19,492 records were appeared through the search (Scopus: 10,836, PubMed/MEDLINE: 1915, and WoS: 6741). After removing 8257 duplicates, titles and abstracts of the remained records were screened. Subsequently, the full-text of 244 relevant articles were assessed for eligibility. Finally, 14 eligible articles were selected and assessed in terms of quality.

### Studies characteristics

The review included 5 cross-sectional (including 2 baseline data of trials), 4 case-control, and 5 cohort studies conducted in Mexico (k = 1), Indonesia (k = 1), Iran (k = 2), Iraq (k = 1), India (k = 2), USA (k = 2), Finland (k = 1), China (k = 2), Australia (k = 1), and Canada (k = 1).

Two case-control studies were conducted among GDM compared with healthy pregnant women [16, 17]. Mousa et al. (2017) studied the baseline serum samples of a large RCT conducted among overweight or obese pregnant women who were susceptible to GDM [18]. Three studies were conducted among preeclampsia or hypertensive disorders in pregnancy (HDP) cases [19–21]. In a case-control study by Budhwar et al. (2020), the biomarkers in cord blood samples of pre-term and term babies were measured [22]. Rosendahl examined cord blood of 939 healthy term infants [20]. One study applied in pregnant women with preterm delivery and their infants cord blood [23]. Perichart-Perera et al. examined the biomarkers of women with SGA fetus [24]. More details about the selected studies are presented in Table 1.

**Table 1:** Summary of the observational studies assessed the relationship between serum vitamin D and inflammatory /oxidative biomarkers in pregnant women

First Author, year	Design	Location	Participants	Health condition	Gestational age (week)	Outcome/Exposure	Quality appraisal
Nassr, 2022	Cohort	Baghda, Iraq	n= 80	Healthy	28–39	25-OH-D, CRP	Very good
Otilia Perichart-Perera, 2022	Cohort	Mexico, Mexico City	SGA (n = 14) AGA (n = 63)	SGA	NI	25-OH-D, MDA, TAC, HbA1c	Very good
Lisnawati, 2021	Cross-Sectional	Indonesia, Jakarta	Maternal 25(OH)D (ng/mL): Normal (n = 22); Insufficiency (n = 42); Deficiency (n = 6)	Preterm birth	28–34	serum vitamin D of the maternal and umbilical cord, umbilical cord IL-6, and serum CRP in premature infants	Satisfactory
Yaqiong, 2020	Cross-Sectional	China, Lianyungang City	Case (n = 110) Control (n = 100)	GDM	NI	25(OH)D, hs-CRP, TNF-α in maternal serum	Good
Jin, 2020	Prospective Birth Cohort	China, Hefei	n = 2479	Healthy	second trimester (14–27)	25(OH)D, hs-CRP in maternal serum	Good
Budhwar, 2020	Case-Control	India, Varanasi	Cord blood of pre-term babies (n = 20) Cord blood of term babies (n = 20)	Pre-term birth	The case group: 27–36 wk The control group: >37 wk	25(OH)D, S100A8, HMGB1, TLR2, NF-kappa B in cord blood serum	Satisfactory
Mousa, 2017	Baseline Data of a RCT	Australia, Melbourne	n = 102	Overweight or obese pregnant women at high-risk of GDM	12–15	25(OH)D, IL-6, in maternal serum	Very good
Rosendahl, 2017	Baseline data of a Trial	Finland, Helsinki	cord blood of healthy term infants (n = 939)	Healthy	at birth	25(OH)D, hs-CRP, in cord blood plasma	Very good
Adela, 2017	Cross-sectional	India, Hyderabad	Case: n = 30 Control: n = 30	HDP	Case:33–37 Control: >24	25(OH) D3, 1,25(OH)2 D3, 25(OH)D2, and 1,25(OH)2 D2, IL-1β, IL-2, IL-4, IL-5, Il-6, IL-7, IL-8, IL-9, IL-10, IL-12, IL-13, IL-15, IL-17, IFN-γ, TNF-α in maternal serum	Very good
Pourghassem Gargari, 2016	Case-Control	Iran, Tabriz	Case (n = 40) Control (n = 40)	PE	≥ 31	25(OH)D, hs-CRP, MDA, TAC in maternal serum	Good
Haidari, 2016	Case-Control	Iran, Ahvaz	Case (n = 45) Control (n = 45)	GDM	20–30	25(OH)D, hs-CRP, TNF-α in maternal serum	very good
Bobbitt, 2015	Cohort	USA, Detroit	n = 178	Healthy	13–28 (The mean gestational age at 25-OHD measure was 9.9 ± 3.6 wk, and mean gestational age at inflammation measurement was 21.1 ± 3.7 wk)	hs-CRP, IL-6, IL-10, IL-1 α and TNF-α in the maternal serum	Very good
McManus, 2014	Case-control	Canada, London	Case (n = 36) Controls (n = 37)	GDM	31	25(OH)D, CRP, PAI-1, IL-8 TNF-α, resistin in maternal and cord blood serum	Satisfactory
Xu, 2014	Retrospective cohort	USA, Pennsylvania	case (n = 100) control (n = 100)	PE	35	25(OH)D, IL-6 in maternal plasma	Good

n: number, wk: week, GDM: gestational diabetes mellitus, NGD: non-GDM, NI: Not Indicated, 25(OH)D: *25*-​*hydroxyvitamin D*3, hs-CRP: high-sensitivity C-reactive protein, IL: Interleukin, TNF-α: Tumor Necrosis Factor-alpha, NF-kappaB: nuclear factor kappa B, MDA: Malondialdehyde, TAC: Total antioxidant capacity, IFN-γ: Interferon Gamma, CRP: C-reactive protein, TGF-β: Transforming growth factor-beta, PAI-1: plasminogen activator inhibitor-1, ELISA: enzyme-linked immunosorbent assay, RIA: Radioimmunoassay, HDP: hypertensive disorders in pregnancy, GH: gestational hypertension, PE: preeclampsia, EC: eclampsia, CLIA: competitive chemiluminescent immunoassays, UPLC/APCI/HRMS: ultra-high performance liquid chromatography/ *Atmospheric pressure chemical ionization*/ *Human Resources Management System*

### Serum 25(OH)D and maternal outcomes

In the study of Yaqiong et al., a significant lower serum concentration of 25(OH)D among GDM cases in comparison to the control group was observed [25]. Moreover, serum concentrations of 25(OH)D had a negative correlation with hs-CRP levels (r= -0.24, p < 0.001), and the risk of developing GDM increased by higher levels of hs-CRP (OR 1.40, 95% CI 1.09–1.80, p = 0.008) and TNF-α (OR 1.22, 95% CI 1.07–1.41, p = 0.004) [25]. In another study by Haidari et al., healthy participants had a negative association between serum concentration of 25(OH)D and hs-CRP (r= -0.44, p = 0.003) [16]. A negative relationship between the levels of 25(OH)D and hs-CRP among healthy pregnant women has been reported by Jin et al., as well [26]. The results of evaluating the association between serum vitamin D metabolites concentration and the levels of cytokine/chemokine in pregnant women with hypertensive disorders showed a positive correlation between 25(OH)D and TNF-α (r = 0.34, p = 0.010), macrophage inflammatory protein-1α (MIP-1α) (r = 0.35, p = 0.008) and MIP-1β (r = 0.27, p = 0.034) and also, a positive correlation between 1,25(OH)2D and IL-9 (r = 0.26, p = 0.047), IL-17 (r = 0.26, p = 0.045), INF-γ (r = 0.27, p = 0.039), and MIP-1β (r = 0.33, p = 0.010) [27]. Bobbitt et al., represented that 25(OH)D had a negative association with IL-1β (p = 0.002) [28]. A large cohort study among preeclamptic women showed that vitamin D deficiency and IL-6 concentration had an independent positive correlation with the risk of preeclampsia [21]. It means that the relationship between vitamin D deficiency and preeclampsia was not as a result of activating the inflammation [21]. The concentrations of 25(OH)D negatively associated with IL-6 among overweight or obese women who were at high risk for GDM (r= -0.20, p = 0.048) [29]. A case-control study by Pourghassem Gargari et al., among women with preeclampsia and their healthy counterparts showed lower serum 25(OH)D concentration in preeclampsia participants which was positively correlated with TAC levels (beta = 0.43, p = 0.010) [19].

### Serum 25(OH)D and fetal outcomes

In a cohort study on 939 healthy infants with the aim of assessing the relationship between the levels of vitamin D in cord bloods and the status of inflammatory markers, a direct correlation between 25(OH)D and hs-CRP serum levels (B coefficient 1.00, 95% CI 1.00-1.01, p = 0.018) was observed [20]. Budhwar et al., found that the insufficient concentration of cord serum 25(OH)D in pre-term infants were related to the impaired balance of inflammation in their mother’s placenta [30]. A case-control study on GDM mothers (n = 36) and controls (n = 37), found that maternal serum concentration of 25(OH)D was associated with IL-8 (r = 0.52, p = 0.020) and TNF-α (r = 0.48, p = 0.030) in a significant positive manner among GDM cases [31].

### Risk of bias assessment

Based on NOS, the included studies were ranked as very good (k = 7), good (k = 4), and satisfactory (k = 3) in terms of quality (Table 1).

## Discussion

Overall, an adverse association between the levels of 25(OH)D and hs-CRP in GDM cases [16, 25], healthy pregnant women [32] and infants’ cord blood [20] was reported by some studies in this systematic review. In addition, vitamin D deficiency concurrent with altered levels of cytokines/chemokines has been shown in pregnant women with hypertensive disorders [33]. On the contrary, one of the included studies proposed a positive association between the levels of 25(OH)D and IL-8 and TNF-α among pregnant women with GDM [31]. Similarly, another other studies suggested an adverse relationship between 25(OH)D concentrations and IL-6 [18] and IL-1β [28].

Evidence suggests a controversial association between vitamin D status and pro-inflammatory markers which can be due to the design of the included studies which made it impossible to extract the causal relationships. Another reason can be attributed to the differences in the time and methods of measuring vitamin D status and the type of inflammatory biomarkers.

In general, some cytokines/chemokines along with other molecules including growth factors and hormones are produced by immune and placental cells to maintain pregnancy [34]. In this regard, cytokines profile is of great importance [34]. It is suggested that vitamin D has a role as immune modulator and cytokine production at the maternal-fetal interface [35]. Vitamin D can switch the immune system from pro inflammatory to anti-inflammatory responses [36, 37]. Although 25(OH) D and inflammation relationship could be U-shaped [38].

Some studies reported that pregnant women experience a rise in the levels of calcitriol during the first trimester which stayed in the same level until the end of pregnancy [39, 40]. However, the results of some longitudinal studies proved that the increment of calcitriol continues until the end of pregnancy [41, 42]. The increasing trend of calcitriol during first trimester might confirm its role in regulating immune function and inflammatory system. The results of a study have shown that calcitriol inhibited cultured trophoblast cells, which are treated with TNF-α to imitate a pro-inflammatory condition, from producing pro-inflammatory cytokines such as IL-6, INF-γ, and TNF-α [34, 43].

A mechanism that have been suggested for the effect of vitamin D on the inflammation is its role in the down-regulation of pro-inflammatory markers including IL-6 and TNF-α [44, 45]. It has been also proposed that vitamin D can affect the production of progesterone-induced blocking factor (PIBF) which poses immunomodulatory role [46, 47]. Another mechanism for the effect of vitamin D on reducing inflammatory status is its role in inhibiting cyclooxygenase-2 (COX-2) expression, increasing 15-prostaglandin dehydrogenase (15-PGDH) and subsequently stop prostaglandin (PG) pathway or reducing the production of PG. Vitamin D can also reduce the expression of EP and FP PG receptor and thus disrupt the PG signaling [48–50].

In this systematic review, one of the included studies showed that the levels of TAC and 25(OH)D had a positive association with each other in preeclamptic women [19]. Another systematic review declared that vitamin D supplementation increased TAC levels [51]. TAC levels represents all the antioxidants in the body and their cumulative effect [52].

Vitamin D anti-oxidant capacity and, therefore, its contribution to prevent protein and lipid peroxidation and DNA damage has been recognized [14]. Active form of vitamin D affected the expression of some genes through its nuclear receptors [14]. Therefore, vitamin D deficiency can be a reason for the complications that are related to the oxidative stress [14]. The result of a study suggested that vitamin D deficiency in pregnant women can disturb vasoconstrictor (thromboxane B-2) and vasodilator (6-keto prostaglandin F-1 alpha) eicosanoids balance and subsequently resulted in the endothelial dysfunction and increased risk of adverse pregnancy outcome [53].

Vitamin D is known to have a role in reducing oxidative stress status through deactivating nuclear transcription factor kB (NF-kB) as a result of elevating the expression of IkB and decreasing the phosphorylation of IkB-α [54–56]. Therefore, vitamin D can disrupt pathways that are dependent on NF-κB [57, 58] and reduce free radicals production.

The effect of vitamin D on oxidative stress status can also be explained by its role in stimulating the production of Nrf2 and subsequently anti-oxidant enzymes [59]. Moreover, Vitamin D reduces oxidative stress through lessening free radicals production and disrupting pathways that are dependent on NF-κB [57, 58]. It also regulates the generation of cellular glutathione and superoxide dismutase [60, 61], suppresses the expression of nicotinamide adenine dinucleotide phosphate (NADP) enzyme [62], and prevents the advanced glycation end products to be accumulated [63], which may in turn suppress oxidative stress.

### Strengths and limitations of the study

According to the authors’ knowledge, this is the first systematic review that assessed the observational evidence regarding the association between vitamin D status and inflammatory and oxidative biomarkers in pregnant women. This systematic review met all the principles of PRISMA, such as conducting a comprehensive search strategy and assessing the quality using standard tools. The present systematic review has also some limitations like variability in study design, the characteristics of the participants, the methods of measuring vitamin D, inflammatory and oxidative stress status, time of launching the studies, and outcomes measurement. these conditions made it impossible to do meta-analysis and reach to conclusive results. Therefore,, assessing the association between vitamin D status and the levels of inflammation and/or oxidative stress biomarkers needs further works regarding the nature of different diseases.

## Conclusions

According to the systematic review of the observational literature, evidence supports the hypothesis that there is a negative association between 25(OH)D levels and hs-CRP among healthy pregnant women, those with GDM, and in cord blood serum. Calcidiol levels have inverse association with serum IL-8 and TNF-α in GDM subjects. Moreover, 25(OH)D concentration of pregnant women has a positive association with TAC levels. Further observational studies are required to have a better understanding of the modulatory role of vitamin D on inflammatory and oxidative status of pregnant women and their offspring.

### Electronic supplementary material

Below is the link to the electronic supplementary material.

Supplementary Material 1

## Data Availability

Not applicable.

## Declarations

### Supporting information

S1. Search terms.

## Abbreviations

NOS: Newcastle-Ottawa Scale
hs-CRPL: high-sensitivity C-reactive protein
IL-1β: Interleukin-1beta
IL-6: Interleukin-6
TNF-α: tumor necrosis factor- alfa
IL-8: Interleukin-8
MIP: macrophage inflammatory protein
GDM: gestational diabetes mellitus
TAC: total antioxidant capacity
HDP: hypertensive disorders of pregnancy
ROS: reactive oxygen species
IUGR: intra uterine growth restriction
PRISMA: Preferred Reporting Items for Systematic Reviews and Meta-analyses
TGF-β: transforming growth factor-beta
IFN-γ: interferon-gama
MDA: malondialdehyde
GSH: glutathione
SOD: superoxide dismutase
MIP-1α: macrophage inflammatory protein-1α
