# Supplementary material for: Vitamin D and biomarkers of inflammation and oxidative stress among pregnant women: a systematic review of observational studies
Source: BMC Immunol. 2023 Oct 27;24:41. doi: 10.1186/s12865-023-00577-w (PMC10612223; doi:10.1186/s12865-023-00577-w)
Supplement: Supplementary file 1 — Supplementary Material 1 [file 12865_2023_577_MOESM1_ESM.docx]

S1. **Search terms**

**Title: Vitamin D and biomarkers of inflammation and oxidative stress among pregnant women: a systematic review of observational studies**

**The search terms for PubMed was as follow:**

(pregnan*[tiab] OR gestation[tiab] OR “child bearing”[tiab] OR childbearing[tiab] OR gravidity[tiab] OR “intrauterine pregnancy”[tiab] OR “labor presentation”[tiab] OR “labour presentation”[tiab] OR “pregnancy maintenance”[tiab] OR “pregnancy trimesters”[tiab]) AND (“vitamin d”[tiab] OR 25(OH)D[tiab] OR “25(OH) D”[tiab] OR “25 (OH) D”[tiab] OR “25 hydroxy vitamin D 3”[tiab] OR 25(OH)D3[tiab] OR “25(OH) D3”[tiab] OR “25 (OH) D3”[tiab] OR “1, 25 dihydroxy vitamin D 3”[tiab] OR calcifediol[tiab] OR calcitriol[tiab] OR Calciol[tiab] OR “Vitamin D 3”[tiab] OR “Vitamin D3”[tiab] OR Cholecalciferol*[tiab] OR “Hydroxyvitamins D”[tiab] OR Hydroxycholecalciferol[tiab] OR Calciferol*[tiab] OR “Vitamin D 2”[tiab] OR “Vitamin D2”[tiab] OR Ergocalciferol[tiab] OR “25 Hydroxyvitamin D 2”[tiab] OR “25 Hydroxyergocalciferol”[tiab] OR “25-Hydroxyvitamin D2”[tiab] OR “25 Hydroxyvitamin D2”[tiab] OR 25-Hydroxycalciferol[tiab] OR “25 Hydroxycalciferol”[tiab] OR “(3 beta,5Z,7E)-9,10-Secocholesta-5,7,10(19)-trien-3-ol”[tiab] OR Calciferols[tiab] OR “25-hydroxyvitamin D”[tiab] OR “24,25 dihydroxyvitamin D”[tiab] OR “25 hydroxyvitamin D”[tiab] OR “9,10 secocholesta 5,7,10(19) trien 23 yne 1,3,25 triol”[tiab] OR “9,10 secocholesta 5,7,10(19) trien 23 yne 3,25 diol”[tiab] OR “9,10 secocholesta 5,7,10(19),16 tetraen 23 yne 1,3,25 triol”[tiab] OR “9,10 secocholesta 5,7,10(19),22 tetraene 1,3,25,26 tetrol”[tiab] OR “ascorbic acid plus fluoride plus retinol plus vitamin D”[tiab] OR “calcium carbonate plus ferrous fumarate plus vitamin D”[tiab] OR “calcium phosphate dibasic plus ferrous sulfate plus manganese sulfate plus nicotinic acid plus riboflavin plus thiamine plus vitamin D”[tiab] OR “9,10-Secoergosta-5,7,10(19),22-tetraene-3 beta,25-diol”[tiab] OR Ercalcidiol[tiab] OR Tachystin[tiab] OR Dihydrotachysterin[tiab] OR Calcamine[tiab])

**The search terms for Web of Science (WoS) was as follow:**

(TS=(pregnan*) OR TS=(gestation) OR TS=(“child bearing”) OR TS=(childbearing) OR TS=(gravidity) OR TS=(“intrauterine pregnancy”) OR TS=(“labor presentation”) OR TS=(“labour presentation”) OR TS=(“pregnancy maintenance”) OR TS=(“pregnancy trimesters”)) AND (TS=(“vitamin d”) OR TS=(25(OH)D) OR TS=(“25(OH) D”) OR TS=(“25 (OH) D”) OR TS=(“25 hydroxy vitamin D 3”) OR TS=(25(OH)D3) OR TS=(“25(OH) D3”) OR TS=(“25 (OH) D3”) OR TS=(“1, 25 dihydroxy vitamin D 3”) OR TS=(calcifediol) OR TS=(calcitriol) OR TS=(Calciol) OR TS=(“Vitamin D 3”) OR TS=(“Vitamin D3”) OR TS=(Cholecalciferol*) OR TS=(“Hydroxyvitamins D”) OR TS=(Hydroxycholecalciferol) OR TS=(Calciferol*) OR TS=(“Vitamin D 2”) OR TS=(“Vitamin D2”) OR TS=(Ergocalciferol) OR TS=(“25 Hydroxyvitamin D 2”) OR TS=(“25 Hydroxyergocalciferol”) OR TS=(“25-Hydroxyvitamin D2”) OR TS=(“25 Hydroxyvitamin D2”) OR TS=(25-Hydroxycalciferol) OR TS=(“25 Hydroxycalciferol”) OR TS=(“(3 beta,5Z,7E)-9,10-Secocholesta-5,7,10(19)-trien-3-ol”) OR TS=(Calciferols) OR TS=(“25-hydroxyvitamin D”) OR TS=(“24,25 dihydroxyvitamin D”) OR TS=(“25 hydroxyvitamin D”) OR TS=(“9,10 secocholesta 5,7,10(19) trien 23 yne 1,3,25 triol”) OR TS=(“9,10 secocholesta 5,7,10(19) trien 23 yne 3,25 diol”) OR TS=(“9,10 secocholesta 5,7,10(19),16 tetraen 23 yne 1,3,25 triol”) OR TS=(“9,10 secocholesta 5,7,10(19),22 tetraene 1,3,25,26 tetrol”) OR TS=(“ascorbic acid plus fluoride plus retinol plus vitamin D”) OR TS=(“calcium carbonate plus ferrous fumarate plus vitamin D”) OR TS=(“calcium phosphate dibasic plus ferrous sulfate plus manganese sulfate plus nicotinic acid plus riboflavin plus thiamine plus vitamin D”) OR TS=(“9,10-Secoergosta-5,7,10(19),22-tetraene-3 beta,25-diol”) OR TS=(Ercalcidiol) OR TS=(Tachystin) OR TS=(Dihydrotachysterin) OR TS=(Calcamine))

**The search terms for Scopus was as follow:**

( TITLE-ABS-KEY ( pregnan* )  OR  TITLE-ABS-KEY ( gestation )  OR  TITLE-ABS-KEY ( "child bearing" )  OR  TITLE-ABS-KEY ( childbearing )  OR  TITLE-ABS-KEY ( gravidity )  OR  TITLE-ABS-KEY ( "intrauterine pregnancy" )  OR  TITLE-ABS-KEY ( "labor presentation" )  OR  TITLE-ABS-KEY ( "labour presentation" )  OR  TITLE-ABS-KEY ( "pregnancy maintenance" )  OR  TITLE-ABS-KEY ( "pregnancy trimesters" )  AND  TITLE-ABS-KEY ( "vitamin d" )  OR  TITLE-ABS-KEY ( 25  ( oh )  d )  OR  TITLE-ABS-KEY ( "25(OH) D" )  OR  TITLE-ABS-KEY ( "25 (OH) D" )  OR  TITLE-ABS-KEY ( "25 hydroxy vitamin D 3" )  OR  TITLE-ABS-KEY ( 25  ( oh )  d3 )  OR  TITLE-ABS-KEY ( "25(OH) D3" )  OR  TITLE-ABS-KEY ( "25 (OH) D3" )  OR  TITLE-ABS-KEY ( "1, 25 dihydroxy vitamin D 3" )  OR  TITLE-ABS-KEY ( calcifediol )  OR  TITLE-ABS-KEY ( calcitriol )  OR  TITLE-ABS-KEY ( calciol )  OR  TITLE-ABS-KEY ( "Vitamin D 3" )  OR  TITLE-ABS-KEY ( "Vitamin D3" )  OR  TITLE-ABS-KEY ( cholecalciferol* )  OR  TITLE-ABS-KEY ( "Hydroxyvitamins D" )  OR  TITLE-ABS-KEY ( hydroxycholecalciferol )  OR  TITLE-ABS-KEY ( calciferol* )  OR  TITLE-ABS-KEY ( "Vitamin D 2" )  OR  TITLE-ABS-KEY ( "Vitamin D2" )  OR  TITLE-ABS-KEY ( ergocalciferol )  OR  TITLE-ABS-KEY ( "25 Hydroxyvitamin D 2" )  OR  TITLE-ABS-KEY ( "25 Hydroxyergocalciferol" )  OR  TITLE-ABS-KEY ( "25-Hydroxyvitamin D2" )  OR  TITLE-ABS-KEY ( "25 Hydroxyvitamin D2" )  OR  TITLE-ABS-KEY ( 25-hydroxycalciferol )  OR  TITLE-ABS-KEY ( "25 Hydroxycalciferol" )  OR  TITLE-ABS-KEY ( "(3 beta,5Z,7E)-9,10-Secocholesta-5,7,10(19)-trien-3-ol" )  OR  TITLE-ABS-KEY ( calciferols )  OR  TITLE-ABS-KEY ( "25-hydroxyvitamin D" )  OR  TITLE-ABS-KEY ( "25 hydroxyvitamin D" )  OR  TITLE-ABS-KEY ( "9,10 secocholesta 5,7,10(19) trien 23 yne 1,3,25 triol" )  OR  TITLE-ABS-KEY ( "9,10 secocholesta 5,7,10(19) trien 23 yne 3,25 diol" )  OR  TITLE-ABS-KEY ( "9,10 secocholesta 5,7,10(19),16 tetraen 23 yne 1,3,25 triol" )  OR  TITLE-ABS-KEY ( "9,10 secocholesta 5,7,10(19),22 tetraene 1,3,25,26 tetrol" )  OR  TITLE-ABS-KEY ( "ascorbic acid plus fluoride plus retinol plus vitamin D" )  OR  TITLE-ABS-KEY ( "calcium carbonate plus ferrous fumarate plus vitamin D" )  OR  TITLE-ABS-KEY ( "calcium phosphate dibasic plus ferrous sulfate plus manganese sulfate plus nicotinic acid plus riboflavin plus thiamine plus vitamin D" )  OR  TITLE-ABS-KEY ( "9,10-Secoergosta-5,7,10(19),22-tetraene-3 beta,25-diol" )  OR  TITLE-ABS-KEY ( ercalcidiol )  OR  TITLE-ABS-KEY ( tachystin )  OR  TITLE-ABS-KEY ( dihydrotachysterin )  OR  TITLE-ABS-KEY ( calcamine ) )
